# Recognizing Bedside Events Using Thermal and Ultrasonic Readings

**DOI:** 10.3390/s17061342

**Published:** 2017-06-09

**Authors:** Danielsen Asbjørn, Torresen Jim

**Affiliations:** 1 UiT—The Arctic University of Norway, 8505 Narvik, Norway; 2 UiO—University of Oslo, 0373 Oslo, Norway; jimtoer@ifi.uio.no

**Keywords:** bedside event detection, fall detection, thermal array, ultrasonic sensor, artificial intelligence, classification

## Abstract

Falls in homes of the elderly, in residential care facilities and in hospitals commonly occur in close proximity to the bed. Most approaches for recognizing falls use cameras, which challenge privacy, or sensor devices attached to the bed or the body to recognize bedside events and bedside falls. We use data collected from a ceiling mounted 80 × 60 thermal array combined with an ultrasonic sensor device. This approach makes it possible to monitor activity while preserving privacy in a non-intrusive manner. We evaluate three different approaches towards recognizing location and posture of an individual. Bedside events are recognized using a 10-second floating image rule/filter-based approach, recognizing bedside falls with 98.62% accuracy. Bed-entry and exit events are recognized with 98.66% and 96.73% accuracy, respectively.

## 1. Introduction

Investigations show that approximately 20% of all registered falls occur in intervention or hospital settings [1,2]. These falls amplify an already challenging situation, cognitively or physically, of the individual falling. In addition, the healthcare cost for both patients and their family and the hospitals increases [3]. In nursing homes, intervention settings and hospitals, most falls occur when the patient tries to get out of or in to bed [4,5,6,7,8]. While seniors in hospital or intervention settings contribute to around 20% of all registered falls, approximately 50% of all falls resulting in hospitalization occur in the home environment by community-dwelling seniors [2]. Out of these falls, around 30% occur in the bedroom [9].

Even though call alarms are widely available, the usage of these alarms when a fall occurs are limited due to the individual either not being able to activate the alarm after the fall, not choosing to use it, or not wearing it during the accident. The importance of being able to recognize falls, independently of a manually triggered alarm, becomes even more evident when we know that falls resulting in an inability to get up after the fall are strongly associated with serious injuries, admission to hospitals, and subsequent moves into long-term care [10].

Systems using sensors attached to the floor [5], body [11] or bed [12] are common. It is however not clear that such equipment reduces the severity of the falls or the number of falls [13,14,15]. The presence of multiple bed exit alarm devices in the market are however evidence that clinicians are searching for methods to become aware of patients trying to get out of bed.

Bedside event recognition is one approach being employed clinically and explored in research to provide staff with warnings that patients with an increased risk of falls (often older patients with cognitive impairment and multiple comorbidities) are about to get up from the bed or chair without the required supervision or assistance [11]. How effective the bed-exit alarms are in terms of reducing falls is however not clear. In hospital wards, the fall rate is relatively low compared to what is observed in nursing homes or sub-acute wards with cognitively impaired people [16]. An older study of 70 individuals (*n* = 70) in a geriatric hospital ward found no reduction in falls or fall related injuries using bed exit alarms [14]. Similarly, a more recent and larger (*n* = 27.672) cluster randomized control trial did not find a reduction in fall rate, even though there was an increased use of bed exit alarms [13]. This study used one or two weight-sensitive pads to determine a bed exit event. Shee et al. [16] did a single cohort study evaluating the effectiveness of a sensor alarm in reducing falls for patients (*n* = 34) with cognitive impairment. They used a bed/chair mat to detect movement and triggered alarms based on threshold values. A repeated measure (A-B-A) single cohort design was used to examine the effectiveness in respect to fall outcomes of the electronic sensor bed/chair alarm. The alarm system was found to be effective, feasible and acceptable as a fall prevention strategy for cognitively impaired patients. A significant decrease in number of falls in the intervention period was observed compared to pre- and post-intervention periods.

It is likely that the lack of evidence of bed-exit alarms as a valuable tool for reducing falls is due to evaluations of the installations of the devices as a single intervention tool only. It seems however that the bed-exit system and protocol need to be tuned differently, based on cognitive capabilities of the individual being monitored. In 2009 Dykes et al. [17] reported on a larger six-month study showing a positive correlation between the actual number of falls and the awareness of fall risk, both in hospital settings and intervention settings. By raising the awareness of fall risk of the individuals, the number of falls was reduced. With individuals being cognitively impaired, different approaches may be more effective as reported by Shee et al. in [16]. They reported on the use of bed-exit alarms in a ward with the cognitively impaired (i.e., having a Mini-Mental State examination score: 12.2). The bed-exit alarms were used to signal nurses about individuals that were getting out of bed. We expect bed-exit systems to become very valuable when such systems not only recognize the situations signaling the intention of the individual to rise up from bed or sit down in bed, but also are able to alter the fall risk awareness of the individual accordingly. Danielsen et al. [18] provide a discussion and design of a fall risk awareness protocol (FRAP) that is suitable in this context. The FRAP combines data from different sensors, both ambient and wearables, and feeds the data into a fall risk probability engine. The fall risk probability engine combines the actual readings with historical data and current health information to create a fall risk probability score. The fall risk probability score is then used for alarming health personnel about the event about to take place and to alert the patient about the potential hazardous situation. Fall risk awareness is in this context addressing the bed-exiting individual along with everyone with formal or informal responsibilities in respect to care of the person being monitored [17,19,20].

The approach presented in this paper builds upon the work presented by Danielsen in [21]. We investigate bedside events, but do this by using other evaluating approaches towards identifying location and posture. The approach towards identifying a heat imprint has been altered to support changing ambient temperatures. While [21] used a fixed temperature threshold to recognize a heat imprint, the approach presented here uses a thermal background removal algorithm and an improved heat disposal algorithm for this purpose. Finally, all algorithms for recognizing the bedside events have been improved, resulting in an approach that has promising properties.

## 2. Related Work

Numerous approaches on automated recognition of individuals leaving bed, entering bed, falling out of bed and bed occupancy in general have been presented for use in hospitals. Madokoro et al. [12] developed a prototype of plate-shaped sensors using piezoelectric films and a monitoring system consisting of microprocessor boards with wireless modules to capture data from sensors. They used six sensors, strategically placed in the bed, to detect movement. The amplified and noise-cancelled signals were fed into Counter Propagation Networks (CPNs)—an algorithm based on self-organizing maps. The approach recognized seven distinct behaviors with a recognition rate between 52.4% and 88.1%. The mean recognition rate was 75%.

In [11], Capezuti et al. used two sensor setups to recognize bed-exits: a dual sensor system using an infrared beam detector combined with a pressure sensitive sensor, and a single pressure sensitive sensor. Fourteen nursing home residents participated in the study for 256 nights. In addition to evaluating accuracy of the approaches by themselves, they evaluated nuisance alarm frequency, and false alarms activated by the system. They found the dual sensor bed-exit alarm to be more accurate than the pressure-sensitive alarm in identifying bed-exiting body movements and reducing the incidence of false alarms. However, false alarms were not eliminated altogether. Poisson regression modeling was used to recognize alarm conditions.

Ranasinghe et al. [22] developed a battery-less, low power and low cost wearable alternative, the Wearable Wireless Identification and Sensing Platform (WISP). They used a single kinematic sensor. Accelerometer readings combined with strength of the transmitted signal where used to recognize bed exit and entry movements. The approach was tested using two different locations: the sensor attached to the mattress and the sensor attached to sternum. The best sensor location was determined to be over sternum. They reported specificity of 97.5% and sensitivity of 92.8% when recognizing bed entry events, while bed exit events were reported with specificity of 93.8% and sensitivity of 90.4%. We have, based on the results reported, calculated the corresponding accuracy to be 95.18% for bed entry and 92.22% for bed exit using the sternum sensor.

Camera-based approaches have been investigated as well. This is especially true for the Microsoft Kinect Depth platform that offers depth image capabilities. Ni et al. [23] used depth images using the Kinect to recognize the “patient gets up from the bed” with an accuracy rate of 98%. The approach used 240 video samples consisting of 40 positive and 200 negative samples (each sample was 5–10 s long), cropped from a 30-minute video of four subjects. Rantz et al. [24] did a combined experimental and field study using a similar approach. The Microsoft Kinect was installed in six hospital rooms in Progressive Care, and data was recorded over 24 h per day over a period of eight months. They focused on recognizing three types of falls: falls from a standing position, falls from a bed, and rolling out of a bed. The study did not report anything on accuracy, but reported a sensitivity of 92% and 95% specificity on 100 weeks’ worth of data, and a false-positive rate of 11 per month per room.

The number of approaches towards fall detection, activity or bedside event recognition using thermal arrays is however very limited. Further, the approaches found during our search for system using thermal arrays were mostly experimental in nature and performed in a controlled environment. The only field try was done by Sixsmith et al. in [25] that used a 16 × 16 thermal array to recognize falls. The system recognized 30% of all falls. More recently, Mashiyama et al. [26] have reported on an 8 × 8 low-cost thermal array mounted in a hallway ceiling for detecting falls. They used a k-Nearest Neighbor (k-NN) algorithm as classifier on a dataset consisting of 20 consecutive frames to detect falls with a relatively high accuracy of approximately 95%. Rafferty et al [27] reports on a similar scenery using a wide-angle 31 × 32 thermal array as sensory input, and with an accuracy of 68% using computer vision techniques to detect falls. Thermal imaging has been tested for use in toilet rooms as well. Kido et al. [28] used 400 heat imprint patterns to create a discriminating equation using data from a 47 × 48 thermal array. They reported to recognize falls in a toilet room with an accuracy of 97.8%. Wong et al. [29] reported on a faint detection surveillance system that was able to detect if a person was laying down with an accuracy of up to 96.15%, dependent on light conditions.

Even though some work has been done on using thermal arrays for fall detection, none has been addressing bedside falls or bedside events. The only approach we have been able to find on doing this is [21]. In [21], Danielsen reports an accuracy of 96.9% on bedside falls, 90.0% on bed entry events, and 93.9% on bed-exit events using an 80 × 60 thermal array and an ultrasonic sensor.

## 3. Materials and Methods 

In [21], Danielsen presents the hardware setup used to collect, pre-process and recognize bedside events. The hardware setup consisted of a BeagleBone Black (BBB) processing platform [30], a FLIR Lepton 80 × 60 thermal array [31], and a Maxbotix ultrasonic sensor [32], all integrated into a single device and mounted in the ceiling giving it a vertical viewpoint. He also developed a bed detection and layout algorithm recognizing the bed from the floor using thermal readings alone [21].

The FLIR Lepton 80 × 60 Thermal Array [31] is a long-wave infrared (LWIR) camera module with 51° Horizontal Field and 63.5° Diagonal Field of View. It captures infrared radiation input in its nominal response wavelength band (from 8 to 14 microns) and outputs a uniform thermal image using the Serial Peripheral Interface Bus (SPI) with an 8.6 frame rate. Each frame is transferred as a sequence of integer numbers that represent the temperature in each pixel of the frame. The thermal sensitivity in the array is 0.05 °C. The sensor is controlled using a two-wire I2C-like serial-control interface. The FLIR Lepton was mounted in the FLIR Breakout Board [33].

The Maxbotix Ultrasonic Sensor MB-1202 I2CXL-MaxSonar EZ0 [32] use I2C two-wire serial control for access and control, and is able to do up to 40 readings per second. Distance readings range from 25 cm up to around 220 cm in our setting.

**Figure 1:**
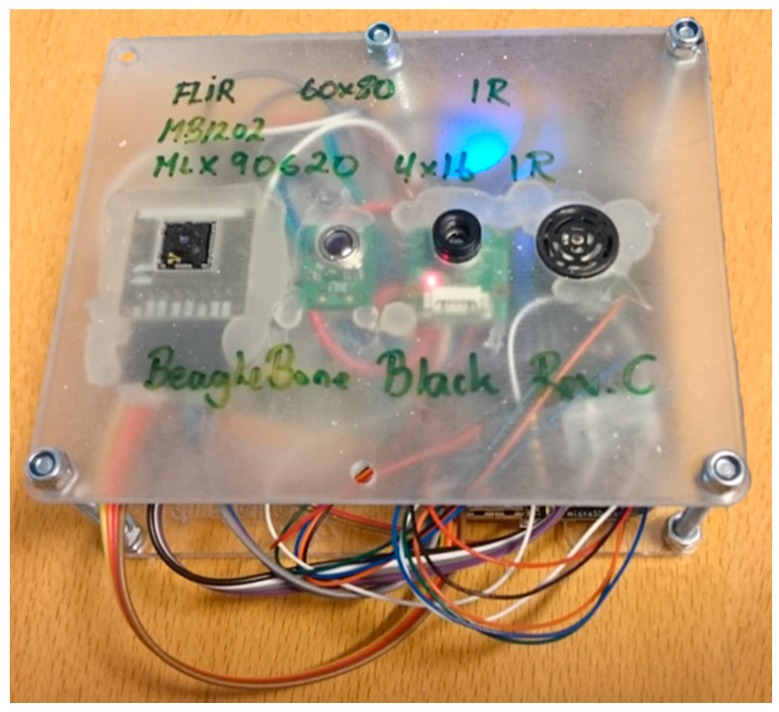
Experimental setup.

Both the FLIR Lepton Breakout Board and the Maxbotix sensor were interfaced to the BBBs I2C-bus. In addition, the FLIR sensor was interfaced to the BBBs SPI-bus for data transfer. The device containing the BBB processing unit and the sensor used in the experiment was 8 × 12 × 3 cm. This prototype device is shown in Figure 1 where the FLIR thermal sensor is visible on the left in the FLIR Breakout Board while the Maxbotix ultrasonic sensor is located on the right. The two other sensors in the middle of the device were not used in the experiment.

The experiment presented in [21] produced a set of raw data that was recorded. The features used in this paper are extracted using this set. The raw data in [21] was captured once every second and identified as a frame. Each frame consisted of 4801 distinct values; 4800 thermal readings representing the 80 × 60 thermal array, and a single distance reading. The distance reading was in the form of centimeters from the ceiling mounted device to the closest reflecting object, while thermal readings were represented as integer numbers. Each integer represented a reading from a single point of the 80 × 60 thermal sensor with a sensitivity of 0.05 °C. During the experiment presented in [21], 8032 frames was recorded.

**Figure 2:**
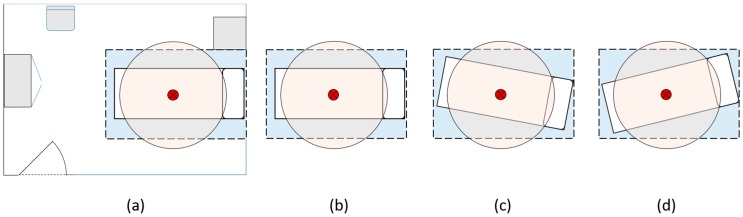
Bed positions and layout of room showing location of the ceiling-mounted device as the small dark red point, the FLIR-sensors field of view as the square blue area, and the ultrasonic sensor area as the semi-transparent circular area: (**a**) Room layout; (**b**) Bed position 1; (**c**) Bed position 2; (**d**) Bed position 3.

The data were recorded at the UiT nursing school in Narvik, Norway. Figure 2a shows the layout of the hospital bedroom used during the experiment. The bed used was an ordinary adjustable hospital bed with rails. The bed was altered into three positions during the experiment to verify the applicability of the approach used for bed outline recognition. The positions are shown in Figure 2b–d. The dark red point over the bed marks the location of the ceiling-mounted sensor and processing device. The FLIR sensor registers thermal readings within the blue square area, while the ultrasonic sensor registers distance readings within the circular area.

### 3.1. Extracting Features

The heat signature of a person is detected using a background subtraction algorithm. The algorithm uses frame differencing on the raw data. The 10 frames used to recognize the bed layout are used to determine the background. Each pixel in every frame is summed and then averaged into an average pixel value for this pixel location; see (1).

(1) $$B\left(x,y\right)=\frac{1}{N}\overset{N}{\underset{i=1}{\sum }}V_{i}\left(x,y\right)$$

*N* is the number of frames used for calculating the average, $V_{i}\left(x,y\right)$ is the sensor value read in frame *i* in the point (x, y). B(x,y) represents the mean background temperature in point (x,y). After the background in a point B(x,y) is calculated, it is subtracted from the value of the current position in the current frame $V_{f}\left(x,y\right)$, thus the heat impression left in a point (x,y) in a single frame $H_{f}\left(x,y\right)$ is:

(2) $$H_{f}\left(x,y\right)=\;\left|\;V_{f}\left(x,y\right)-B\left(x,y\right)\;\right|$$

**Figure 3:**
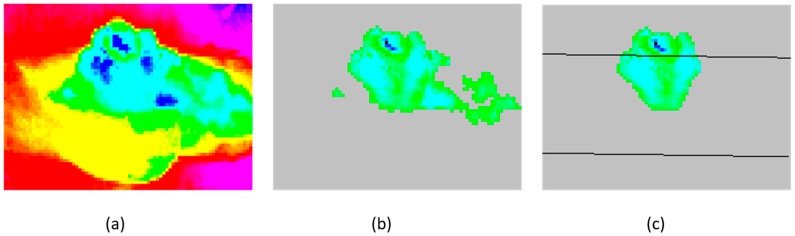
Background heat removal and heat disposal algorithms: (**a**) Frame with person in bed; (**b**) Same as (**a**), but with applied background heat removal algorithm; (**c**) Same as (**b**), but with applied heat disposal algorithm and superimposed representation of bed.

All $H_{f}\left(x,y\right)$ exceeding a threshold value, in our experiment defined as approximately 1 °C, is interpreted as a heat impression in point (x,y) of the actual frame. In Figure 3, (1) has been applied to *N* consecutive frames of thermal readings without heat impressions generating B(x,y) for every point. Then (2) is applied, using the actual reading $V_{f}\left(x,y\right)$ in Figure 3a, creating $H_{f}\left(x,y\right)$ for every point. Figure 3b shows $H_{f}$ of the current frame.

### 3.2. Feature Overview

Due to the BeagleBone Black’s (BBBs) limited processing power, it was preferable to do some pre-processing on the data collected by the thermal sensor to reduce the processing power needed to recognize locations and postures. The bed used in the experiment was an ordinary adjustable bed with a thick duvet and head cushion. During the experiment, the participants in the study were not instructed on how to use the bed, duvet or head cushion. Based on these conditions, the size of a heat imprint, both in and out of the bed, was of interest. However, heat impressions left in the bed or bed linen after the individual had moved would be registered as well; see Figure 3b. This situation would occur whenever an individual moved or exposed some part of the bed or bed linen that had been in contact with the body. Assuming the temperature readings of the body are different from the surroundings, the heat imprint in the exposed area would behave uniformly, decreasing or increasing temperature steadily until it reaches an equilibrium with the surroundings. The background subtraction algorithm processed on every frame makes it possible to identify this situation and act upon it. When an area is recognized as a potential residual heat area, the heat disposal algorithm is executed on the current frame to cancel out the readings from the area containing residual heat. When equilibrium with surroundings has been reached, the heat disposal algorithm addressing the residual heat area terminates. This filtering mechanism is executed on the sensor data, prior to determining the size of heat imprints. Consequently, the heat readings ($P_{fmax\_in}$ and $P_{fmax\_out}$) do not include residual heat as shown in Figure 3c.

Covering the body using the duvet would significantly alter the heat imprint, compared to not using it. Further, the warmest part of the body observed from a vertical viewpoint would most likely be the head of an individual, and the head would normally not be covered by the duvet or the head cushion. Thus, the head of an individual was assumed to be a good indicator on where the body was posed. A clearer interpretation of this would be possible if the maximum temperature inside and outside the bed boundaries was made available ($T_{fmax\_in}$ and $T_{fmax\_out}$).

The heat imprint by itself might not be sufficient in terms of recognizing posture and location. Parts of the body may be obstructed from thermal readings due to the use of bed linen or situations where part of the body cannot be observed due to other objects obstructing the thermal reading, e.g., a person laying partly under the bed after a fall. Getting a distance reading to the closest reflecting object and adding information to identify posture had to be included ($D_{f}$). Finally, a metrics for signaling changes between frames ($M_{f}$) was desirable since it would be an indicator of change of posture, location or even an event. Table 1 gives an overview of the features extracted for this purpose.

Frames without heat impressions are used to filter out the background of the frames with individuals in. The features are extracted once every second. The frame features in Table 1 are used by machine learning algorithms to determine location and posture, as presented in Table 2.

**Figure 4:**
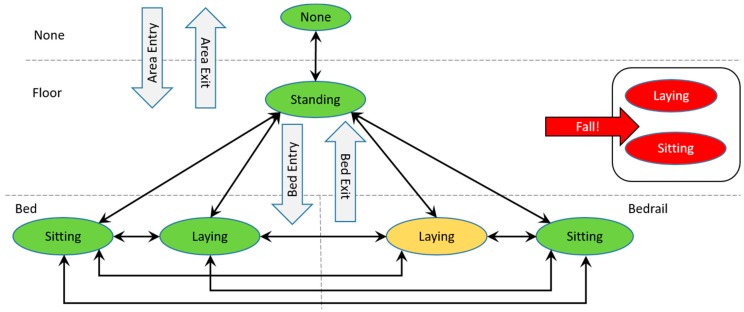
Transitions and events identification.

The event detection algorithm uses the features in Table 2, along with the corresponding indication of changes between frames $M_{f}$ and the distance reading $D_{f}$, analyzing *N* consecutive frames leading up to the current frame recognizing transitions, as shown in Figure 4.

Classification of location $L_{f}$ is performed using $P_{fmax\_in}$, $P_{fmax\_out}$, $T_{fmax\_in}$, and $T_{fmax\_out}$ as input to a classification algorithm. When location has been classified, $P_{fmax\_in}$, $P_{fmax\_out}$, $T_{fmax\_in}$, $T_{fmax\_out}$, $L_{f}$, and $D_{f}$ are used for classifying the posture $PO_{f}$. Events are recognized by analyzing the transitions between stable locations and postures using a number of consecutive frames. The heat disposal algorithm suppresses residual heat left in the bed when an individual leaves the bed.

In Figure 4, the location where the body heat signature is detected is separated using dashed lines (None, Floor, Bed, and Bedrail). The oval shapes indicate postures (None, Standing, Sitting, Laying) recognized in the different locations. The solid arrows between postures show how postures change. Events are represented as filled arrows with an event label. Any change of posture into Laying or Sitting posture on Floor is interpreted as a Fall-event. Other events recognized are Area Entry/Exit and Bed Entry/Exit. Laying on the Bedrail is considered possibly hazardous.

### 3.3. Interpreting Location and Posture 

In terms of recognition, a clear definition of what a location is and how it is interpreted, and what a posture is and how this is interpreted needs to be established, see Table 3.

The classification algorithms may produce incorrect classifications in some frames of location and/or posture that could influence the event recognition. To remove noise from the dataset used for event recognition, some simple tests are executed. These tests address incorrect classification of location and posture, e.g., a heat imprint being classified as $L_{f}=(Bed\;|\;Bedrail)$ and $PO_{f}=Standing$. Such frames are considered erroneous and removed from the data set. Further, if either location or posture is classified as *None*, the other attribute is altered to *None* and the frame forwarded to event processing.

### 3.4. Event Recognition

**Figure 5:**
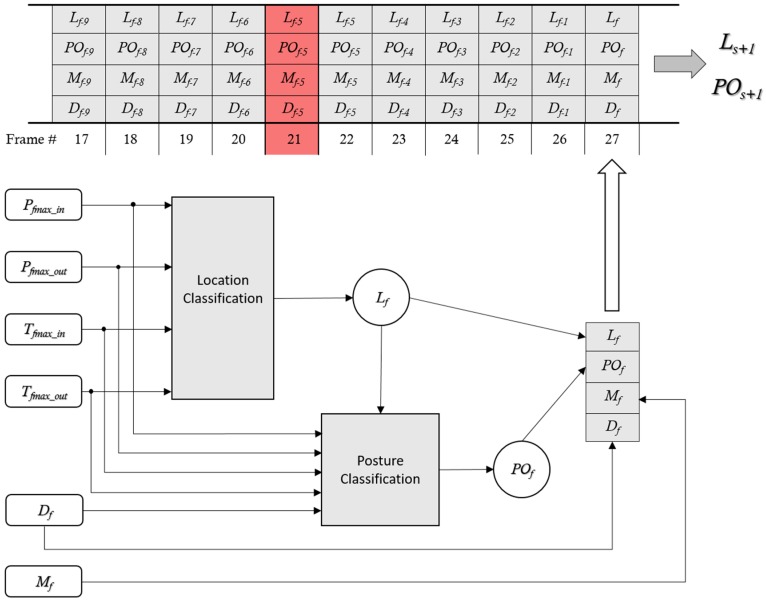
Detecting a new stable posture $PO_{s+1}$ and stable location $L_{s+1}$ using a floating window.

Event recognition is done by analyzing *N* consecutive frames in terms of location ($L_{f}$) and posture ($PO_{f}$) in a frame *f*, with corresponding distance readings ($D_{f}$) and indications of changes between previous frame and current frame ($M_{f}$). $L_{s}$ denotes the previous recognized stable location, and $PO_{s}$ denotes the previous recognized stable posture. For a location or posture to be denoted stable, the location or posture has to be unchanged for at least *N* consecutive frames. Figure 5 shows how the classification of location and posture in a frame are processed and forwarded into a floating window along with distance readings and indication of changes in between frames. The floating window used for recognizing a potentially new stable posture $PO_{s+1}$ and stable location $L_{s+1}$ is dynamic. In Figure 5, the floating windows are defined to have *N* = 10 accepted classifications of location and posture. In the figure, frame 21 is determined as erroneous and consequently dismissed. This may be caused by a distance reading that is not compatible with the classified location or posture. When *N* consecutive frames have been processed, resulting in a new stable location and/or posture, the newly detected stable location $L_{s+1}$ and posture $PO_{s+1}$ are compared to the previously detected stable location $L_{s}$ and posture $PO_{s}$. If a change of stable posture and/or stable location is found, an event condition may have been detected. In any case, the change of stable location and/or posture is updated. Finally, the next frame is fetched and a new sequence of recognizing a new stable location and/or posture is started.

The Fall event is recognized as a change from any stable location $L_{s}$ or posture $PO_{s}$ resulting in a situation where the individual is recognized in a new stable location/posture with $L_{s+1}=Floor$ and $PO_{s+1}=(Laying|Sitting)$, in *N-1* consecutive frames. *N = 10* is used for detecting the Fall event. $M_{f}$ is analyzed due to a fall tending to be a physical stressful incident, which significantly alters the heat-impression in between frames. Finally, if an individual falls out of bed, the residual heat left in the bed should steadily decrease and be detected by the heat disposal algorithm. Consequently, an abrupt decrease of heat impression pixels in the bed should be observable while the number of heat impression pixels outside the bed should abruptly increase and then become stable.

The Area Entry and Area Exit events are recognized as situations in which a heat imprint totally leaves or enters the thermal sensory area. Recognition of Area Entry and Area Exit use *N* = 5 for this purpose. Area Entry is defined as an event in which $L_{s}=None$ and $PO_{s}=None$, and $L_{s+1}\neq None$ and $PO_{s+1}\neq None$. The Area Exit is defined as $L_{s}\neq None$ and $PO_{s}\neq None$, and $L_{s+1}=None$ and $PO_{s+1}=None$.

The Bed Entry event is recognized as a change from any $L_{s}=(Floor|None)$ resulting in a situation in which the individual is recognized in a new stable location/posture, with either $L_{s+1}=Bed$ and $PO_{s+1}=(Laying|Sitting$) or $L_{s+1}=Bedrail$ and $PO_{s+1}=Sitting$, in *N-1* consecutive frames. The Bed Exit event is recognized as a change from any $L_{s}=(Bed|Bedrail)$, resulting in a situation in which the individual is recognized in a new stable location/posture with $L_{s+1}=Floor$ and $PO_{s+1}=(Laying|Sitting|Standing$), or $L_{s+1}=None$ and $PO_{s+1}=None$, in *N-*1 consecutive frames. The recognition of the Bed Entry and Exit events uses *N =* 10 for this purpose. In addition, in each frame leading up to the current stable $L_{s}$ and $PO_{s}$, $D_{f}$ is evaluated to ensure that frames with incorrectly recognized $L_{f}$ and $PO_{f}$ are dismissed. $M_{f}$ is further analyzed due to a Bed Entry and Bed Exit often being an incident, which significantly alter the heat-impression in between frames.

## 4. Results

In this paper we focus on presenting how different classification algorithms perform as well as the final event recognition. The dataset, a total of 8032 frames, was manually labeled with the correct location and posture. The manually labeled data set was split into two separate sets; a learning set and a test set, using a congruential random generator with a predefined seed selecting frames targeted for the different sets. The learning set was defined to be 20% of the 8032 frames, i.e., 1606 frames, while the test set was the remaining 80%, i.e., 6426 frames.

Learning has been applied on a separate machine, while testing has been executed on the BeagleBone Black to ensure the ability of this platform to execute the algorithms in a hospital, intervention or home setting.

### 4.1. Recognizing Location

In [21], Danielsen showed how a decision tree may be used to recognize the location $L_{f}$ with 94.5% correctly recognized instances on the training set using a 10-fold cross validation test mode. In the first part, $P_{fmax\_in}$, $P_{fmax\_out}$, $T_{fmax\_in}$, and $T_{fmax\_in}$ were used to train recognition of the correct location $L_{f}$. We applied this to implementations of Multilayer Perceptron (MLP), k-Nearest Neighbor (k-NN), and a Decision Tree.

#### 4.1.1. Multilayer Perceptron Model

**Figure 6:**
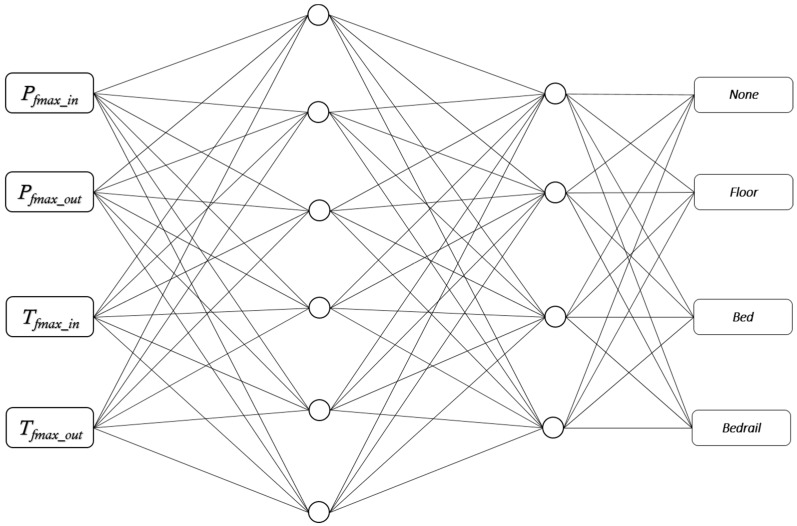
Multilayer Perceptron (MLP) representation with two hidden layers for recognizing location (*L_f_*).

The Multilayer Perceptron used four input channels and had two hidden layers—6 × 4, and four outputs as illustrated in Figure 6. The momentum of the learning was set to 0.1, while the learning rate was set to 0.2. The generation used a 10-fold cross validation test mode, resulting in 92.03% correctly recognized instances (1478) and 7.97% incorrectly recognized instances (128) in the learning set. Applying the classification on the test set of 6426 instances resulted in 91.86% correctly recognized instances (5903) and 8.14% incorrectly recognized instances (523).

#### 4.1.2. k-Nearest Neighbor

An implementation of k-NN with k = 7 using the Euclidian distance to identify the nearest neighbors was used. The generation used a 10-fold cross validation test mode, resulting in 91.78% correctly recognized instances (1474) and 8.22% incorrectly recognized instances (132) in the learning set. When executing this classification on the test set of 6426 instances, it resulted in 91.91% correctly recognized instances (5906) and 8.09% incorrectly recognized instances (520).

#### 4.1.3. J48 Decision Tree

A C4.5 [34] decision tree using the J48 implementation of WEKA was generated. The generation used a 10-fold cross validation test mode, resulting in a pruned tree with 90.6% correctly recognized instances (1455) and 9.4% incorrectly recognized instances (151) in the learning set. When executing this classification on the test set of 6426 instances, it resulted in 91.38% correctly recognized instances (5872) and 8.62% incorrectly recognized instances (554). The tree had 42 leaves.

#### 4.1.4. Findings

The results using the selected algorithms are similar in terms recognition, as shown in Table 4.

By analyzing the actual matrices on what was being correctly processed, a slightly different interpretation emerged. Based on our data, it seemed like the MLP was best suited for recognizing situations with no heat imprint (location = None), while the J48 Decision Tree was better suited for recognizing heat impressions on Floor and in Bed. k-NN had better results for recognizing Bedrail locations. Table 5 gives an overview of these findings.

However, the overall changes by optimizing are marginal. The recognition rate of using a MLP is 91.9%, and optimizing the recognition rates using different algorithms to target different locations only increased the total recognition rate to 93%.

### 4.2. Recognizing Posture

In [21], Danielsen used the correctly recognized frames, in terms of recognizing location $L_{f}$, as the input for posture recognition. A decision tree was used to recognize the correct posture $PO_{f}$ with 98% correctly recognized instances on the learning set using a 10-fold cross validation test mode. The approach presented here is different. The learning set used for classifying location was also used for classifying posture. $P_{fmax\_in}$, $P_{fmax\_out}$, $T_{fmax\_in}$, $T_{fmax\_in}$, $D_{f}$, and a manually correctly tagged $L_{f}$ were used to train recognition of the correct posture $PO_{f}$. We applied this to implementations of MLP, k-NN, and a Decision Tree.

#### 4.2.1. Multilayer Perceptron Model

The MLP used six input channels, had two hidden layers—6 × 4, and four outputs. The momentum of the learning was set to 0.1 while the learning rate was set to 0.2. The generation used a 10-fold cross validation test mode, resulting in 81.63% correctly recognized instances (1311) and 18.37% incorrectly recognized instances (295) in the learning set. When executing this classification on the test set of 6426 instances it resulted in 80.39% correctly recognized instances (5166) and 19.61% incorrectly recognized instances (1260).

#### 4.2.2. k-Nearest Neighbor

An implementation of k-NN with k = 3 using the Eucledian distance to identify the nearest neighbors was used. The generation used a 10-fold cross validation test mode, resulting in 84.12% correctly recognized instances (1351) and 15.88% incorrectly recognized instances (255) in the learning set. When executing this classification on the test set of 6426 instances it resulted in 84.13% correctly recognized instances (5406) and 15.87% incorrectly recognized instances (1020).

#### 4.2.3. J48 Decision Tree

A C4.5 [34] decision tree using the J48 implementation of WEKA was generated. The generation used a 10-fold cross validation test mode, resulting in a pruned tree with 82.25% correctly recognized instances (1321) and 17.75% incorrectly recognized instances (285) in the learning set. When executing this classification on the test set of 6426 instances it resulted in 83.82% correctly recognized instances (5386) and 16.18% incorrectly recognized instances (1040). The tree had 80 leaves.

#### 4.2.4. Findings

The results using the selected algorithms were similar in terms of how good the recognition was, as shown in Table 6.

The differences in recognition rate were small. Both the J48 Decision Tree and k-NN produced very similar results with a recognition rate of respectively 83.82% and 84.13%. By analyzing the confusion matrices of the classification, see Table 7, it became clear that the differences were marginal and an optimization of the classifications using different algorithms for different classification purposes would only increase the overall recognition rate to 85.06%.

The posture recognition of Sitting and Standing contributes significantly to lower the overall recognition rate. When recognizing the Sitting posture, more than 16.6% of the frames were recognized as Laying, independent of classification algorithm. A similar and more serious observation is related to the recognition rate of the Standing posture (only applicable in a Floor location). More than 27.7% of all frames containing a Standing posture were classified incorrectly.

The Standing posture is challenging to classify based on the approach used for classification. We used the size of the heat imprint made by an individual, in or out of bed, along with maximum temperature readings, in and out of bed, to classify location. Ideally, the distance sensor should cover the exact same area as the thermal array, but this is not the case, as visualized in Figure 2. The classification algorithms have no information on whether the heat imprint is located within the area covered by the distance sensor. Consequently, it is only the size of the heat imprint along with maximum temperatures that contribute when classification of posture is being done in such frames. This seems to be the major contributing factor to this misclassification.

Location was classified as None, Bed, Bedrail and Floor. Further, the Sitting posture was applicable to all, except None. The major parts of Sitting postures that are misclassified are classified as Laying. Investigation into the actual frames where posture is misclassified shows that most of these misclassifications are related to the individual moving around in the bed, adjusting the duvet or head cushion, turning around, etc. and thereby influencing the distance readings. The frames capturing this are the major source of incorrect Sitting posture classification.

To verify that the explanations were correct, a new classification test was run using the identical learning and test sets, but this time classifying location and posture in one single classification. We used WEKA and a J48 Decision Tree for this purpose. Table 8 gives the confusion matrix of this verification using the test set of 6426 instances, and verifies that our explanations are correct; 76.69% of all frames in the test set were correctly classified.

### 4.3. Preparing for Event Recognition

In terms of deciding upon which classification approach to use for recognizing location, the Multilayer Perceptron and k-NN, using k = 7, produced close to identical results. However, considering the limitations of the dataset and the simple nature of the instance-based learning used in k-NN, the Multilayer Perceptron approach using two hidden layers, as shown in Figure 6, was preferred. In terms of recognizing posture, k-NN approach using k = 3 had best results. To verify that this was the best-suited combination, a cross-validation test was run on all presented combinations of AI algorithms for identifying location $L_{f}$ and posture $PO_{f}$. The results of the test confirmed the expectations and are presented in Table 9. The test was executed on the complete dataset (8032 frames), including the set used for learning. The numbers presented in Table 9 are the numbers of correctly recognized location $L_{f}$ and posture $PO_{f}$.

Recognizing location $L_{f}$ and posture $PO_{f}$ yield some erroneous results in our approach. For the selected algorithms, the recognition rate of $L_{f}$ is 91.86% using a MLP. Similarly, the recognition rate using a k-NN algorithm with k = 3 for $PO_{f}$ is 84.13%. The actual correct recognition of both $L_{f}$ and $PO_{f}$ is however only 79.74%, as presented in Table 9.

Both $L_{f}$ and $PO_{f}$ are recognized by using single frames. The event analyses discussed in the next section use a number of consecutive frames to analyze whether an event has happened or not. In such an analysis, the prime assumption is that both $L_{f}$ and $PO_{f}$ are stable, i.e., they are not fluxing and changed in between frames.

### 4.4. Event Recognition Metrics

The dataset used in this paper consists of 8032 frames. These frames represent 28 recordings in which a number of events occur. The observed and manually recorded events are what we call actual events. Programmatically detected events are either true or false. A true event is an actual event that is correctly programmatically detected, while a false event is an event that is recognized programmatically, but no corresponding actual event exists. We define this as:

- True Positive (*TP*): An actual event has occurred, and an event is correctly programmatically recognized. This is a true event.
- False Positive *(FP*): No actual event has occurred, but an event is incorrectly programmatically recognized. This event is a false event.
- True Negative (*TN*): No actual event has occurred, and the algorithm, correctly, does not recognize any event.
- False Negative (*FN*): An actual event has occurred, but the algorithm incorrectly does not recognize the event.

The following metrics are used:

(3) $$Accuracy=\frac{TP+TN}{TP+TN+FP+FN}$$

(4) $$Precision=\frac{TP}{TP+FP}$$

(5) $$Sensitivity=\frac{TP}{TP+FN}$$

(6) $$Specificity=\frac{TN}{TN+FP}$$

(7) False Positive Rate=1−Specifity

(8) False Negative Rate=1−Sensitivity

Accuracy (3) indicates how good the differentiation between recognizing events and not recognizing them are. Precision (4) is the probability of a recognized event being an actual event. Sensitivity (5) is the probability of recognizing all actual events. Specificity (6) is the probability of correctly not recognizing an event when no actual event exists. Finally, the False Positive Rate (7) is the probability of a recognized event, not being an actual event, while the False Negative Rate (8) is the probability of an actual event not being recognized.

### 4.5. Event Recognition

During the experiment, all frames were analyzed. The recordings consisted of 145 events. Out of these, 128 events were correctly recognized, giving a recognition rate of 91.7%. Table 10 gives a detailed overview of the results and Table 11 presents information on accuracy, sensitivity, precision, and specificity, along with the False Positive and False Negative rates of all events.

## 5. Discussion

The recognition of bedside events is very good. This is especially true for the Fall and the Bed Entry events, which results in an accuracy of 98.62% and 98.66%, respectively. The findings are actually slightly better with respect to the Fall event. The two false positives registered in the Fall event are secondary falls happening when the individual is trying to get up after a fall, but fails to do so.

When comparing the results presented here to those presented in [21], the numbers of actual events differ. This is due to differences in the data included in the reported events. Both papers use the same raw dataset, but the event-results presented here include all 8032 frames while [21] does not include its learning set in its evaluation. The learning set of [21] consisted of 15 events and 829 frames.

While Danielsen in [21] only used frames that were correctly recognized in terms of location to be used as a learning set for posture, we use the complete learning set independently of whether location was correctly recognized or not for the actual frame in question. Consequently, the recognition rate of identifying posture in this paper is significantly lower than reported in [21].

This paper also addresses some major issues in [21]. While [21] uses a fixed threshold of 25 °C to identify a heat imprint, we use a thermal background removal algorithm combined with a heat disposal algorithm adapted for this purpose. The correctly recognized locations in [21] using an 829-frame learning set and a J48 decision tree were found to be 94.5%. The similar approach presented in this paper uses a larger learning set of 1606 frames and results in 90.6% recognized locations. While Danielsen [21] used a simple approach working well in a room with around 20 °C, the approach presented in this paper will be more flexible and will work independently of room temperature.

The sensitivity and accuracy of Area Entry and Area Exit events have also increased compared to [21], though still with relatively low sensitivity. The participants in the test were eager and healthy, and they moved relatively fast when being instructed to do something. For example, an instruction like “Stand up, walk around bed” could result in a situation in which the individual raised up from the bed, moved out of the observable area, into the area, turned around and moved out of area again, all within six to ten frames (seconds). Some of the individuals even used the bed as a lever to execute the turns faster. This in turn meant that even though the individuals walked around the bed, their hand was on the bedpost in one frame and not in the next, then on the third frame it was on the bedpost again, in the fourth not, and so on contributing to incorrect recognition of both Area Entry and Area Exit events. This contributed to the number of false negatives in Area Entry and Area Exit as well as the sensitivity of these events. We expect Area Entry and Area Exit events to perform better under testing in an actual environment.

The algorithms for recognizing Bed Entry and Bed Exit have been further developed from those used in [21], and the sensitivity and accuracy of these events have increased. However, the Bed Exit event especially suffers from a lower sensitivity than expected. This is due to the relatively high number of false negatives (events that happened, but were not recognized).

The experiment in [21] was executed in a controlled environment, both in terms of air and room temperature, and without other factors like sunlight reflecting on floor or wall, air conditioning devices, etc. Consequently, the effect on the approach related to such external factors has not been addressed. Further on, the approach assumes a single person within the thermal image.

Using a thermal array in activity or event recognition in this context has historically performed poorly, e.g., Sixsmith et al. [25]. This is however changing. Recently, Rafferty et al [27] reported on the use of a wide-angle 31 × 32 thermal array for detecting falls and reports an accuracy of 68% using computer vision techniques to detect falls. Mashiyama et al. [26] reported on an accuracy of 95% using an 8 × 8 ceiling-mounted infrared array in an experimental setting. Wong et al. [29] reported an accuracy of 86.19% to 96.15% in recognizing faint events, dependent on light conditions. The approach presented in this paper offers both higher accuracy and higher sensitivity than other presented approaches using thermal readings. Table 12 gives an overview of some of the properties of the approaches using thermal arrays for recognizing falls that we have found.

Incorrect or noisy readings from the sensors corrupt data used during classification and influence event recognition. Rather than using conventional methods to reduce noise in the dataset, we have addressed the observed noise directly by developing and applying a background subtraction algorithm and a heat disposal algorithm to make the size of the actual heat impressions closer to the actual heat imprint of the body. The noise has been further reduced by addressing incorrect classification of location and posture, incompatible combinations of location and posture, and by introducing the concept of stable location and posture.

The approach presented preprocesses the distance and thermal readings into five properties ($P_{fmax\_in}$, $P_{fmax\_out}$, $T_{fmax\_in}$, $T_{fmax\_out}$, and $D_{f}$), and one derived, $M_{f}$. The preprocessed properties are used for classification of location $L_{f}$ and posture $PO_{f}$ in a frame *f*. During the preprocessing, a number of possible interesting properties are lost, e.g., whether or not the readings are within both sensors detectable area, whether distance reading is outside thermal array area or the other way around, the actual outline of heat imprint, etc. Including this kind of information in a future dataset would increase accuracy and possibly offer new paths to even better results, opening the path for prediction.

## 6. Conclusions and Future Work

The applicability of the approach presented in this paper is not limited to hospital or intervention settings. In this approach, we are able to recognize the outline of the bed in different positions using a thermal array. The unit containing both processing environment and sensors is small and suited for ceiling mounting. Bedside falls make up approximately 30% of all falls by the elderly in a home environment [9], and the consequences of the inability to get up after a fall are very serious [10]. The approach presented here targets bedside falls specifically along with other bedside events, and does so with high accuracy. Further, the processing device may easily be adapted to signal an alarm to centrals or relatives. This makes the approach presented well suited for use in a home environment in terms of fall detection as well as in hospital or intervention settings. Finally, the approach does not require user interaction, does not use intrusive technology, and represents a minor intervention into the home environment of the elderly.

We have verified the robustness of the approach presented in [21] by evaluating different approaches towards recognizing location and posture and getting similar classification results. In addition, weaknesses have been addressed. We have documented and altered the algorithm detecting the heat imprint from using a fixed threshold to be a dynamically adaptable approach including background heat removal and residual heat disposal. This approach works independently of room temperature. Further on, the event recognition has been properly defined building on the concept of stable location and posture. Noise in the dataset along with detection of incorrect classifications has been addressed as well. Consequently, we present better results on event recognition. We have further analyzed the findings on classification in respect to the datasets used and concluded why certain incorrect classifications occur.

Preventing falls from happening in the first place is the ultimate goal. The approach presented here is able to detect bed entry and bed exit events with a very high degree of accuracy. Recognizing that an individual is about to sit down in the bed or an individual is about to leave the bed is very interesting due to this situation being an intervention point in terms of preventing a fall from happening. We believe it is possible, using the identical non-intrusive sensory setup used in [21] and in this paper, to make good predictions on what actions an individual is about to do. In [18], Danielsen et al. presented a framework for raising fall risk awareness by using feedback mechanisms to signal the elderly as well as everyone with a formal or informal responsibility in terms of the elderly being monitored. By recognizing bedside events, and including recognition of the intention of sitting down or raising up, the approach presented in [18] may well be a first step in preventing falls from happening. Studying this in more detail is part of future work.

## Figures and Tables

**Table 1:** Frame features extracted for further processing.

Feature	Explanation
$P_{fmax\_in}$	The number of heat impression pixels found within boundary of bed in frame *f*.
$P_{fmax\_out}$	The number of heat impression pixels outside the boundary of the bed in frame *f*.
$T_{fmax\_in}$	Maximum temperature registered within boundary of bed in frame *f*.
$T_{fmax\_out}$	Maximum temperature registered outside bed boundaries in frame *f*.
$D_{f}$	The number of centimeters from the ceiling mounted ultrasonic sensor to the closest reflecting object in frame *f.*
$M_{f}$	Number of heat impression pixel changes from the previous frame, *f –* 1, to the current frame *f*, expressed as an integer from 0 indicating no changes. The larger the number, the more changes have occurred.

**Table 2:** Location and posture features.

Feature	Explanation
$L_{f}$	Location of the heat imprint in frame *f*. It is recognized using classification algorithms to be one of Bed, Floor, Bedrail, and None. None indicates that the information available is not sufficient to determine location.
$PO_{f}$	Posture recognized in frame *f* using classification algorithms. Posture is classified as one of the following: Laying, Sitting, Standing, or None. None indicates that the information available is not sufficient to determine posture.

**Table 3:** Interpretation of location $L_{f}$ and posture $PO_{f}$.

Location	Posture	Interpretation
None	None	No heat imprint found in frame. Due to the lack of heat imprint in frame, neither location nor posture can be identified.
Bed	Sitting Laying	The individual is either sitting up in bed or laying in bed. The heat imprint is within bed boundaries.
Bedrail	Sitting Laying	The individual is either sitting on the bed with legs partly outside bed, laying in the bed on the bedrail, or sitting on the floor with parts of the upper body, e.g., arms, in the bed. The latter is interpreted as laying on the bedrail. Laying on bedrail is considered hazardous.
Floor	Standing Sitting Laying	The person is either standing on the floor, sitting on the floor, or laying on the floor

**Table 4:** Recognizing location, *L_f_*, of individual in frame.

Approach	Learning Set		Test Set		Total	
	Correct	Fail	Correct	Fail	Correct	Fail
Multilayer Perceptron	1478	128	5903	523	7381	651
k-Nearest Neighbor	1474	132	5906	520	7380	652
Decision Tree (J48)	1455	151	5872	554	7327	705

**Table 5:** Confusion matrix of location classification by algorithms.

Correct	Fail	None	Bed	Floor	Bedrail	Recogn.	Algorithm
176	15	176	6	8	1	None	Multilayer
1699	133	8	1699	8	117	Bed	Perceptron
2395	219	45	13	2395	161	Floor	
1633	156	1	27	128	1633	Bedrail	
151	40	151	6	32	2	None	k-Nearest
1697	135	1	1697	7	127	Bed	Neighbor
2417	197	28	10	2417	159	Floor	
1641	148	0	38	110	1641	Bedrail	
137	54	137	4	45	5	None	Decision
1702	130	4	1702	17	109	Bed	Tree
2458	159	23	5	2458	128	Floor	
1575	214	0	55	159	1575	Bedrail	

**Table 6:** Recognizing posture, *PO_f_*, of individual in frame.

Approach	Learning		Test		Total	
	Correct	Fail	Correct	Fail	Correct	Fail
Multilayer Perceptron	1311	295	5166	1260	6477	1555
k-Nearest Neighbor	1351	255	5406	1020	6757	1275
Decision Tree (J48)	1321	285	5386	1040	6707	1325

**Table 7:** Confusion matrix of posture classification by algorithms.

Correct	Fail	None	Sitting	Standing	Laying	Recogn.	Algorithm
188	16	188	0	15	1	None	Multilayer
1731	756	0	1731	143	613	Sitting	Perceptron
297	115	1	32	297	82	Standing	
2950	373	0	275	98	2950	Laying	
192	12	192	1	11	0	None	k-Nearest
2013	474	5	2013	56	413	Sitting	Neighbor
251	161	13	82	251	66	Standing	
2950	373	1	337	35	2950	Laying	
190	14	190	0	14	0	None	Decision
1935	552	0	1935	119	433	Sitting	Tree
298	114	1	51	298	62	Standing	
2963	360	0	305	55	2963	Laying	

**Table 8:** Confusion matrix of location and posture classification during a J48 single classification run.

None	Floor			Bedrail		Bed		Recogn.	Location/Posture
	Sit	Stand	Lay	Sit	Lay	Sit	Lay	Rate	
183	5	3	0	3	4	1	5	89.71%	None/None
5	400	48	216	8	56	0	0	54.57%	Floor/Sitting
32	73	167	63	47	32	0	10	39.39%	Floor/Standing
6	175	42	1183	6	22	0	1	82.44%	Floor/Laying
4	28	30	9	1158	52	15	52	85.91%	Bedrail/Sitting
1	56	16	13	69	277	0	22	61.01%	Bedrail/Laying
0	0	2	0	15	7	298	74	75.25%	Bed/Sitting
2	2	14	1	56	8	87	1262	88.13%	Bed/Laying

**Table 9:** Cross-validating AI approaches.

Classification		Correctly Recognized			
Location	Posture	Location	Posture	Loc. & Pos.	Of Total
MLP	MLP	7381	6477	6060	75.45%
MLP	J48	7381	6707	6380	79.43%
MLP	k-NN	7381	6757	6405	79.74%
J48	MLP	7327	6477	6000	74.70%
J48	J48	7327	6707	6336	78.88%
J48	k-NN	7327	6757	6361	79.20%
k-NN	MLP	7380	6477	6057	75.41%
k-NN	J48	7380	6707	6368	79.28%
k-NN	k-NN	7380	6757	6394	79.61%

**Table 10:** Event recognition results.

	Fall	Bed Entry	Bed Exit	Area Entry	Area Exit
**Actual events**	26	26	26	46	21
**Recognized**	28	24	23	42	16
**False Positive**	2	0	1	1	1
**False Negative**	0	2	4	5	6
**True Positive**	26	24	22	41	15
**True Negative**	117	123	126	108	135

**Table 11:** Evaluation of results.

	Fall	Bed Entry	Bed Exit	Area Entry	Area Exit
**Accuracy**	98.62%	98.66%	96.63%	96.13%	95.54%
**Precision**	92.86%	100.00%	95.65%	97.62%	93.75%
**Sensitivity**	100.00%	92.31%	84.62%	89.13%	71.43%
**Specificity**	98.32%	100.00%	99.21%	99.08%	99.26%
**False Positive Rate**	1.68%	0.00%	0.79%	0.92%	0.74%
**False Negative Rate**	0.00%	7.69%	15.38%	10.87%	28.57%

**Table 12:** Thermal array approaches for fall detection.

Paper	Year	Sensor	Size	Res.	Mount	Platform	Acc.	#	Age
[21]	2016	FLIR Lepton + ultrasonic	80 × 60	0.05 °C	Vertical	BeagleBone	96.9%	7	23–53
[25]	2004	Irisys	16 × 16	2 °C	Slanted	PC	30%	28	65–82
[26]	2014	Panasonic Grid-EYE	8 × 8	1 °C	Vertical	Arduino	95%	6	N/A
[27]	2016	Heimann IR L5.0/1.0	31 × 32	0.02 °C	Vertical	PC	68%	N/A	N/A
[28]	2009	Chino Co. TP-L0260EN	47 × 48	0.5 °C	Vertical	PC	97.8%	5	N/A
[29]	2010	FLIR A-20M	320 × 240	0.1°C	Slanted	PC	96.2%	1	70
This	2017	FLIR Lepton + ultrasonic	80 × 60	0.05 °C	Vertical	BeagleBone	98.6%	7	23–53
